# Preventive administration of *Bifidobacterium animalis* subsp. *lactis* HEM20–01 attenuates the development of atopic dermatitis-like inflammation in mice, along with gut microbial and succinate alterations

**DOI:** 10.3389/fimmu.2026.1915780

**Published:** 2026-10-13

**Authors:** Young Su Jang, Hyeongjin Na, Hyo-Jin Lee, Yeojin Bang, Jae-Sang Ryu, Haivin Kim, Dahye Seo, Inseon Sim, Dong Ho Suh, Eun Sung Jung, Yosep Ji, Dong Hyun Kim, Hyun Jin Choi, Jung U. Shin

**Affiliations:** 1 Department of Biomedical Science, Graduate School, CHA University, Seongnam, Republic of Korea; 2 Department of Medicine, CHA University School of Medicine, Seongnam, Republic of Korea; 3 HEM Pharma Inc., Suwon, Republic of Korea; 4 College of Pharmacy and Institute of Pharmaceutical Sciences, CHA University, Seongnam, Republic of Korea; 5 Department of Dermatology, Bundang CHA Medical Center, CHA University School of Medicine, Seongnam, Republic of Korea

**Keywords:** atopic dermatitis, *bifidobacterium animalis* subsp. *lactis* HEM20-01, gut–skin axis, microbiome, succinate

## Abstract

**Background:**

Atopic dermatitis (AD) is a chronic inflammatory skin disorder frequently accompanied by gut dysbiosis and neurobehavioral comorbidities, but the microbial metabolites mediating gut–skin crosstalk remain unclear. We investigated whether preventive administration of *Bifidobacterium animalis* subsp. *lactis* HEM20–01 attenuates the development of AD-like phenotypes and characterized the accompanying gut microbiome, succinate, intestinal, and behavioral alterations.

**Methods:**

HEM20–01 was orally administered to NC/Nga mice beginning 2 weeks before house dust mite (HDM) induction and continued throughout the 4-week induction period. Cutaneous and intestinal inflammation were assessed by histology, gene expression, and multiplex cytokine analysis, and behavior by the open field test. Fecal microbiota composition and metabolite profiles were analyzed by 16S rRNA gene sequencing and gas chromatography–time-of-flight mass spectrometry (GC-TOF-MS)-based metabolomics. Exogenous succinate was administered in an independent MC903-induced dermatitis model to test whether succinate can aggravate these responses.

**Results:**

HDM-induced AD-like mice showed activation of the intestinal succinate receptor 1 (SUCNR1)–tuft cell–IL-25 axis and enrichment of the putative succinate-associated family Rikenellaceae and its genus *Alistipes*, together with elevated fecal and plasma succinate. Preventive HEM20–01 administration attenuated the development of cutaneous and intestinal inflammatory phenotypes. HEM20-01-treated mice also showed a lower relative abundance of putative succinate-associated taxa (Bacteroidaceae, Rikenellaceae, Christensenellaceae, *Bacteroides*, and *Alistipes*), lower circulating succinate levels, reduced activation of SUCNR1–tuft cell–IL-25-related signaling, and attenuated behavioral and hippocampal serotonergic alterations compared with untreated AD-like mice. In a separate MC903-induced dermatitis model, succinate supplementation exacerbated cutaneous and intestinal inflammation.

**Conclusions:**

Preventive HEM20–01 administration attenuates the development of AD-like phenotypes and associated behavioral alterations, accompanied by changes in gut microbiome composition, lower circulating succinate, and intestinal inflammatory signaling.

## 1 Introduction

Atopic dermatitis (AD) is a chronic, relapsing inflammatory skin disorder associated with allergic comorbidities, gut and skin microbiome dysbiosis, and neurobehavioral abnormalities (1, 2). The gut microbiota plays a pivotal role in shaping the host immune system, and the gut–skin axis has emerged as an important modulator of cutaneous inflammation (3, 4). Patients with AD frequently exhibit gut dysbiosis, including a reduction in beneficial taxa such as *Bifidobacterium* (5, 6), and disruption of the gut microbial community in AD has been associated with disease onset and chronicity (7, 8). Accordingly, modulating gut microbial homeostasis with beneficial bacteria, such as *Bifidobacterium* strains, has been explored as a potential therapeutic strategy for AD. For example, *Bifidobacterium longum* CCFM1029 alleviates AD symptoms by enhancing tryptophan metabolism and promoting indole-3-carbaldehyde production, which activates aryl hydrocarbon receptor (AhR)-mediated immune regulation (9). Likewise, *Bifidobacterium animalis* subsp. *lactis* (*B. lactis*) BX-BC08 increases α-ketoglutarate, thereby suppressing Th2 inflammatory responses (10).

Identifying the specific metabolic mediators that link gut dysbiosis to systemic immune modulation is crucial for understanding how the microbiome shapes host immune responses. Among gut-derived metabolites, succinate has emerged as a candidate microbiome-derived inflammatory mediator that functions as a danger-associated metabolic signal (11). Under physiological conditions, gut anaerobes such as *Bacteroides* and *Prevotella* produce succinate, which is rapidly consumed by cross-feeding bacteria and converted into downstream metabolites such as propionate, thereby maintaining low luminal concentrations (12). However, dysbiosis accompanied by expansion of succinate-producing or succinate-associated taxa, such as *Bacteroides* or *Prevotella*, can lead to the accumulation of unconsumed succinate (13, 14). Excess succinate can act as a metabolic alarmin by activating succinate receptor 1 (SUCNR1) on intestinal tuft cells, which release IL-25 and initiate group 2 innate lymphoid cell (ILC2)-dependent type 2 immune circuits (14–16). In AD, circulating succinate was elevated in a murine model of atopic march, in which it amplified type 2 immunity through this circuit (14). Serum succinate was likewise higher in allergic children with AD than in allergic children without AD (14). However, it remains unclear whether gut microbial composition is associated with circulating succinate levels in AD, and whether modulating the gut microbiota is accompanied by changes in circulating succinate and in cutaneous and systemic inflammatory phenotypes.

Here, we investigated whether preventive administration of *B. lactis* HEM20-01 (KCTC14143BP) attenuates the development of house dust mite (HDM)-induced AD-like phenotypes. HEM20–01 was selected on the basis of preclinical data disclosed in a United States patent (17), in which oral administration reduced circulating endotoxin, corticosterone, and proinflammatory cytokine levels in mice. We characterized the accompanying differences in gut microbial composition, circulating succinate, intestinal inflammatory signaling, and behavior. In a separate MC903 model, exogenous succinate was administered to test whether it can aggravate dermatitis-like phenotypes.

## 2 Methods

### 2.1 Ethics approval statement

All procedures were approved by the Institutional Animal Care and Use Committee of CHA University (IACUC-230037) and performed in accordance with institutional guidelines.

### 2.2 Experimental animals

Four-week-old male NC/Nga and C57BL/6 mice were purchased from Orient Bio Inc. (Seongnam, Korea). Mice were co-housed (4~5/cage) under a 12-h light/dark cycle with free access to chow and water, and randomly assigned to groups.

### 2.3 HDM-induced dermatitis mouse model with preventive HEM20–01 administration

HDM-induced dermatitis was induced in NC/Nga mice by topical application of 100 mg Biostir-AD (freeze-dried *Dermatophagoides farinae* extract ointment; Biostir Inc., Kobe, Japan) twice weekly for 4 weeks. For barrier disruption, 200 μL of 4% sodium dodecyl sulfate (SDS) was applied 2 h before each Biostir-AD treatment. Clinical severity was assessed weekly using a modified SCORAD-based scoring system (erythema, edema, excoriation, scaling, each 0–3). HEM20–01 was administered orally by gavage (1×10^9^ CFU/mouse/day in 200 μL PBS) starting 2 weeks before induction until the end of induction; controls received vehicle. Groups were NOR, AD and AD+HEM20-01 (*n* = 9 each). Mice were sacrificed 24 h after the final application.

### 2.4 Succinate supplementation in MC903-induced dermatitis mouse model

C57BL/6 mice received sodium succinate dibasic hexahydrate (Sigma-Aldrich, St. Louis, MO, USA, #S9637; 100 mM) in drinking water beginning 2 weeks before AD induction and continuing throughout the 3-week MC903 treatment period, during which 45 μM MC903 (MedChemExpress, Monmouth Junction, NJ, USA, #HY-10001) or ethanol was applied topically to each ear (20 μL per ear) five times per week. Mice were assigned to three groups (n = 9 per group): topical ethanol vehicle with normal drinking water (EtOH), MC903 with normal drinking water (MC903+H_2_O), and MC903 with 100 mM sodium succinate in the drinking water (MC903+succinate). Drinking solutions were replaced every 2–3 days, and water consumption was monitored at the cage level throughout the supplementation period. Based on cage-level monitoring, the estimated intake was approximately 5.2-5.4 mL/mouse/day, corresponding to approximately 0.52–0.54 mmol of sodium succinate per mouse per day. Body weight was measured at the start of succinate supplementation (−2 weeks), immediately before MC903 induction (0 weeks), and at the end of the induction period (3 weeks) (**Supplementary Figure 3**).

### 2.5 Transepidermal water loss

TEWL was measured weekly under standardized conditions (20 ± 2 °C; 50 ± 5% humidity) using a Tewameter^®^ TM-300 (Courage-Khazaka, Cologne, Germany).

### 2.6 Scratching behavior

Mice were acclimated for 15 min in black acrylic chambers, and scratching behavior was video-recorded for 30 min. A scratch bout was defined as a hind limb movement directed toward the ears, face, or dorsal region, and the total number of bouts was counted by three independent investigators blinded to group allocation.

### 2.7 Open field test

After 30 min habituation, mice were placed individually in a 30 × 30 × 30 cm open-field arena and tracked for 10 min using SMART 3.0 (Panlab, Barcelona, Spain). The center zone was defined as the central 25% of the arena area. The distance travelled in the center zone (as a percentage of the total distance travelled), the time spent in the center zone (as a percentage of the total test duration), and the number of center-zone entries were quantified.

### 2.8 Multiplex cytokine analysis

Cytokines in skin, draining lymph nodes, and plasma were quantified using a Luminex Discovery Assay (R&D Systems, Minneapolis, MN, USA, #LXSAMSM). For lymph node cytokines, single-cell suspensions were stimulated with PMA (100 ng/mL; Sigma-Aldrich, #P1585) and ionomycin (1 μM; Sigma-Aldrich, #I0634) for 4 h at 37 °C.

### 2.9 Histological and immunofluorescence analysis

Tissues were fixed in 4% paraformaldehyde and embedded in paraffin. Sections (5 μm thick) were stained with H&E, toluidine blue, or PAS. For tuft cell analysis, duodenal sections were immunostained with anti-DCLK1 (1:200, Abcam, Cambridge, UK, #ab31704) followed by goat anti-rabbit Alexa Fluor 488 (1:1000, Invitrogen, Carlsbad, CA, #A11008) and counterstained with DAPI (1:5000, Sigma-Aldrich, #D9542). Mast cells, goblet cells, and tuft cells were quantified and normalized to tissue area (mm²). For goblet and tuft cells, quantification was performed within individual villi.

### 2.10 Quantitative reverse transcription PCR

Total RNA was isolated with TRIzol (Invitrogen, #15596026), and qRT-PCR was performed using a real-time PCR system (Bioneer, Daejeon, Korea). Gene expression levels were normalized to GAPDH and calculated using the 2^−ΔΔCT^ method. Primer sequences are provided in **Supplementary Table 1**.

### 2.11 Western blot

Tissues were homogenized in RIPA lysis buffer, and lysates were separated by SDS-PAGE and transferred onto PVDF membranes (Millipore, Burlington, MA, USA, #IPVH00010). Anti-SUCNR1/GPR91 antibody (1:1000, Novus, Centennial, CO, USA, #NBP1-00861) was used as a primary antibody and HRP-conjugated goat anti-rabbit IgG was used as the secondary antibody (GeneTex, Irvine, CA, USA, #213110-01). β-actin served as a loading control. Blots were visualized using Clarity Western ECL Substrate (Bio-Rad, Hercules, CA, USA, #1705061) and captured with an Amersham Imager 680 (GE Healthcare, Chicago, IL, USA).

### 2.12 Total and allergen-specific IgE measurement

Total plasma IgE was quantified by Mouse IgE ELISA kit (Abcam, #ab157718). Der f–specific IgE was measured by ELISA using microplates coated with *Dermatophagoides farinae* extract (JA bio, Suwon, Korea, #PEA-DERF010).

### 2.13 5-Hydroxytryptamine and 5-hydroxyindoleacetic acid measurement

Hippocampal 5-HT and 5-HIAA were quantified by HPLC with electrochemical detection (HPLC-ECD). Hippocampi were homogenized in ice-cold 0.4 M perchloric acid (10 mg tissue/100 μL perchloric acid), clarified, filtered, and injected onto an X-bridge column (XBridge™ C18 5 µm, Waters Corp., MA, USA). 5-HT and 5-HIAA were used as external standards, and peaks were identified by retention time matching. Analyte levels were normalized to tissue weight.

### 2.14 16S rRNA sequencing and metabolomics

Fecal samples (colonic contents) were collected individually from each mouse at the time of sacrifice, immediately snap-frozen in liquid nitrogen, and stored at −80 °C until analysis. Samples were processed without pooling and analyzed as independent biological samples for microbiome and metabolomic analyses. Fecal 16S rRNA gene amplicon sequencing and GC-TOF-MS-based metabolomics were performed as described in the **Supplementary Methods**.

### 2.15 Plasma succinate measurement

Plasma succinate levels were measured using a Succinate Assay Kit (Abcam, #ab204718).

### 2.16 Statistical analysis

Data are presented as mean ± SEM. Normality was assessed using the Shapiro–Wilk test, and homogeneity of variance was evaluated using the Brown–Forsythe test. Comparisons among multiple groups were performed using one-way ANOVA followed by Tukey’s multiple-comparisons test. Longitudinal data were analyzed using two-way repeated-measures ANOVA followed by Tukey’s multiple-comparisons test. Center-zone measures from the open field test were additionally analyzed by analysis of covariance (ANCOVA) with total distance travelled as a covariate, after confirming homogeneity of regression slopes (group × covariate interaction, *P* > 0.4 for all measures). For metabolomic analysis, succinate peak areas were log-transformed for visualization. Overall group differences were assessed using the Kruskal–Wallis rank-sum test, followed by pairwise Wilcoxon rank-sum tests with Benjamini–Hochberg false discovery rate (FDR) correction. Statistical analyses were performed using GraphPad Prism 6.0 (GraphPad Software, San Diego, CA, USA), except for the metabolomic analyses and the ANCOVA, which were performed in R software (v4.3.2) and in Python (v3.12; statsmodels v0.15.0), respectively. A *P* value < 0.05 was considered statistically significant.

## 3 Results

### 3.1 Preventive HEM20–01 administration attenuates AD-like cutaneous and systemic inflammation

AD-like inflammation was induced in NC/Nga mice by topical *Dermatophagoides farinae* extract for 4 weeks. To evaluate the effects of HEM20–01 administration, mice were orally treated with HEM20-01 (1 × 10^9^ CFU/mouse/day) from 2 weeks before AD induction until the end of the induction period (NOR, AD, and AD+HEM20–01 groups). HDM-treated NC/Nga mice exhibited increased modified SCORAD index, transepidermal water loss (TEWL), and scratching behavior, accompanied by epidermal hyperplasia and mast cell infiltration. Oral HEM20–01 significantly attenuated the development of these clinical and histological features, as evidenced by reductions in modified SCORAD index, TEWL, scratching behavior, epidermal thickness, and mast cell infiltration (**Figures 1A–E**).

**Figure 1:**
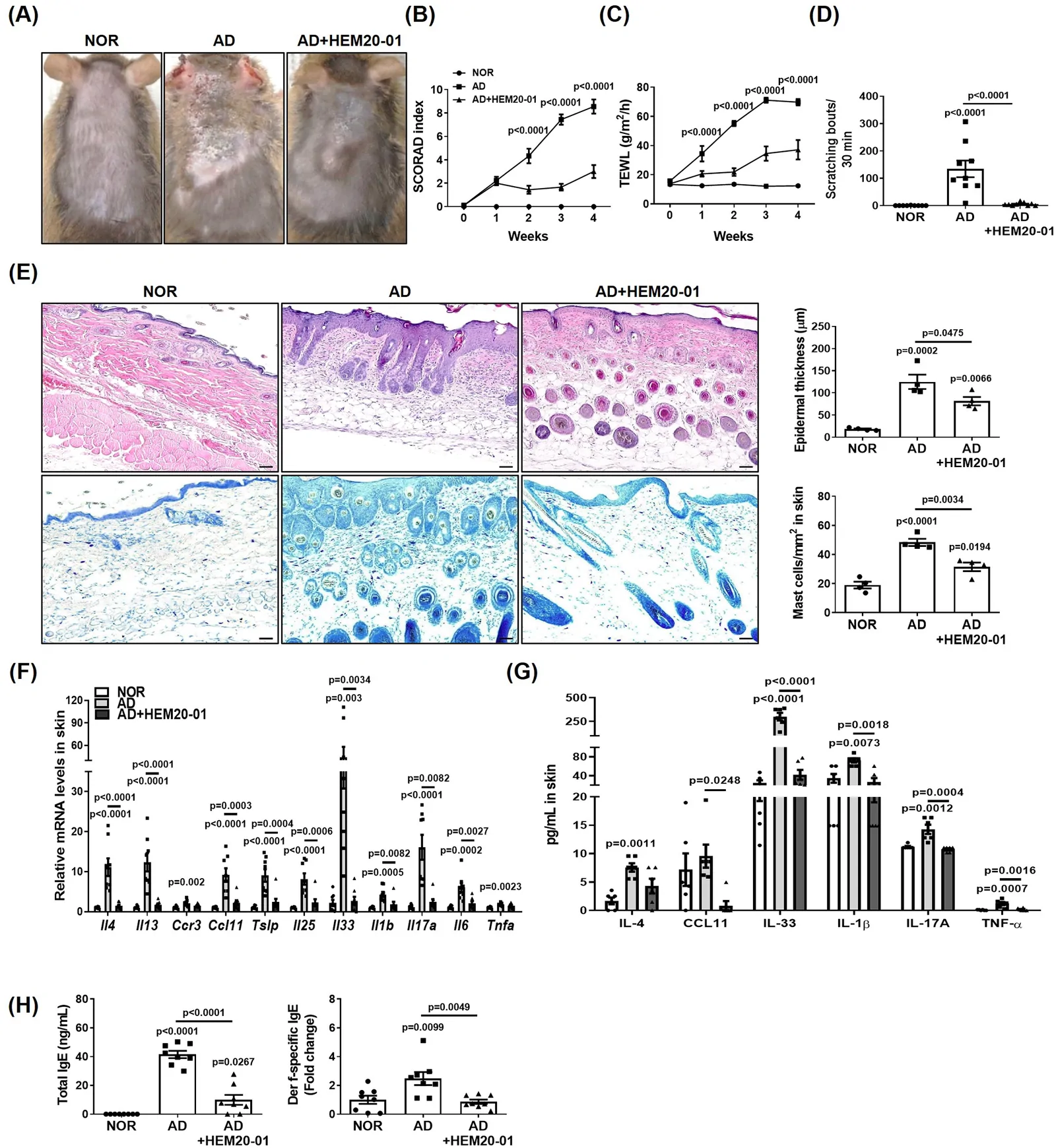
Preventive HEM20–01 administration attenuates the development of AD-like skin inflammation and immune responses. Mice were pretreated with HEM20–01 for 2 weeks prior to the induction of AD-like skin inflammation (NOR, untreated controls; AD, HDM-treated; AD+HEM20-01, HDM-treated with HEM20-01; *n* = 9 per group). **(A)** Representative images of dorsal skin lesions after AD induction. **(B)** SCORAD, **(C)** TEWL, and **(D)** scratching behavior (*n* = 9 per group). Data are presented as mean ± SEM and analyzed using two-way repeated-measures ANOVA in **(B, C)**, followed by Tukey’s test. **(E)** Representative images of H&E and toluidine blue staining in the skin. Scale bars, 50 μm. Quantification of epidermal thickness and mast cell number (*n* = 4 per group). **(F)** mRNA expression of inflammatory genes in the skin (*n* = 8 per group). **(G)** Multiplex cytokine analysis of inflammatory cytokines in the skin (*n* = 6 per group). **(H)** Plasma levels of total IgE and *Der f*–specific IgE (*n* = 8 per group). **(D–H)** Data were analyzed by one-way ANOVA with Tukey’s test. *P*-values are shown.

At the molecular level, the skin of AD-like mice showed marked upregulation of type 2-associated cytokines (*Il4, Il13*), eosinophil/Th2 recruitment-related genes (*Ccr3, Ccl11*), Th2-related epithelial alarmins (*Tslp, Il25, Il33*), Th17-associated cytokine (*Il17a*), and proinflammatory cytokines (*Il1b, Il6, Tnfa*), which were downregulated by HEM20-01 (**Figure 1F**). Consistent with these transcriptional changes, HEM20–01 also reduced the elevated protein levels of IL-4, CCL11, IL-1β, IL-33, IL-17A, and TNF-α in AD-like skin (**Figure 1G**). In parallel, systemic total IgE and *Der f–*specific IgE levels were increased in AD-like mice and significantly reduced by HEM20-01 (**Figure 1H**).

### 3.2 Intestinal inflammatory signatures in AD-like mice are attenuated by HEM20-01

Given emerging evidence for skin-to-gut immune crosstalk (18), we examined whether HDM-induced dermatitis is accompanied by intestinal type 2 inflammation. AD-like mice showed increased goblet and tuft cell numbers, without overt changes in villus-crypt architecture (**Figures 2A, B**). Duodenal SUCNR1 expression was increased at both the protein and mRNA levels, accompanied by upregulation of its downstream effector, *Il25* (**Figures 2C, D**). In parallel, duodenal expression of Th2-related (*Il13*), eosinophil-associated (*Ccr3*, *Ccl11, Prg2*), and proinflammatory (*Il1b*, *Il6*, *Tnfa*) genes was elevated in AD-like mice (**Figure 2D**). Observed intestinal inflammatory signatures were attenuated in HEM20-01-treated mice, which showed lower goblet and tuft cell numbers and reduced expression of inflammatory genes compared with AD-like mice (**Figures 2A–D**). Similarly, mesenteric lymph nodes (mLNs) from AD-like mice exhibited increased IL-5, IL-13, IL-6, and TNF-α levels, which were lower in HEM20-01-treated mice (**Figure 2E**).

**Figure 2:**
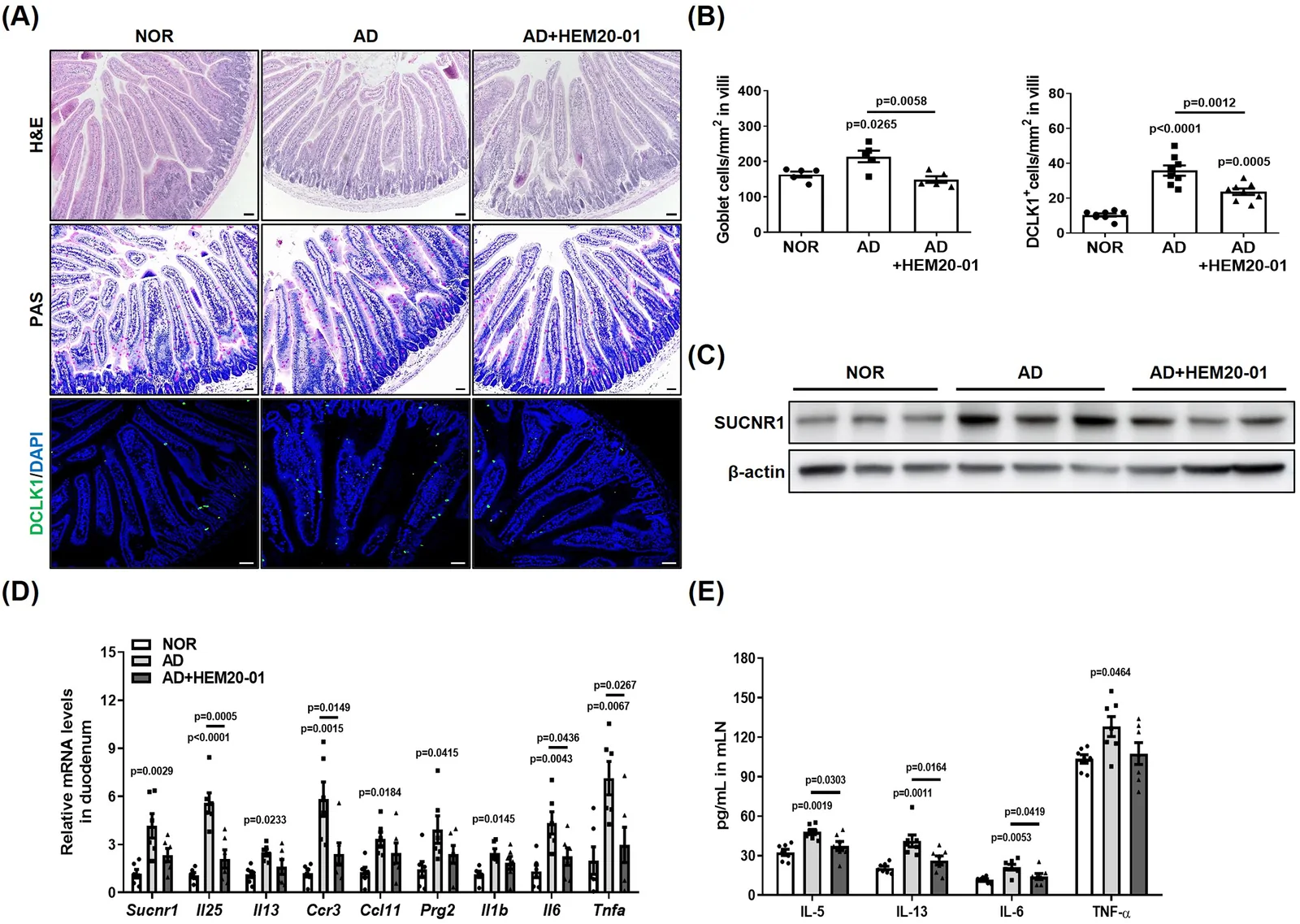
HEM20–01 treatment is associated with attenuated intestinal epithelial and inflammatory alterations. **(A)** Representative images of H&E, PAS, and DCLK1 expression in the duodenum. Nuclei were counterstained with DAPI. Scale bars, 50 μm. **(B)** Quantification of goblet cells (*n* = 5 per group) and DCLK1^+^ cells (*n* = 8 per group) located within villi. **(C)** Western blot analysis of SUCNR1 in the duodenum. β-actin served as a loading control. **(D)** mRNA levels of epithelial and inflammatory markers (*n* = 6 per group). **(E)** Multiplex cytokine analysis of inflammatory cytokines in mLNs (*n* = 7 per group). **(B, D, E)** Data are presented as mean ± SEM and analyzed using one-way ANOVA with Tukey’s test. *P*-values are shown.

### 3.3 Behavioral and hippocampal serotonergic alterations are attenuated in HEM20-01-treated mice

In the open field test (OFT), mice with HDM-induced dermatitis showed reduced center-zone exploration, as indicated by decreases in center-zone distance, center-zone time, and the number of center-zone entries. HEM20-01-treated mice showed greater center-zone exploration than AD-like mice (**Figures 3A, B**; **Supplementary Figure 1A**). Total distance travelled did not differ significantly among groups (*P* = 0.088, **Supplementary Figure 1B**), and the group differences in center-zone distance, time, and entries remained significant after adjustment for total distance travelled (ANCOVA, *P* = 0.0008, 0.0008, and 0.015, respectively), indicating that these differences were independent of overall locomotor activity.

**Figure 3:**
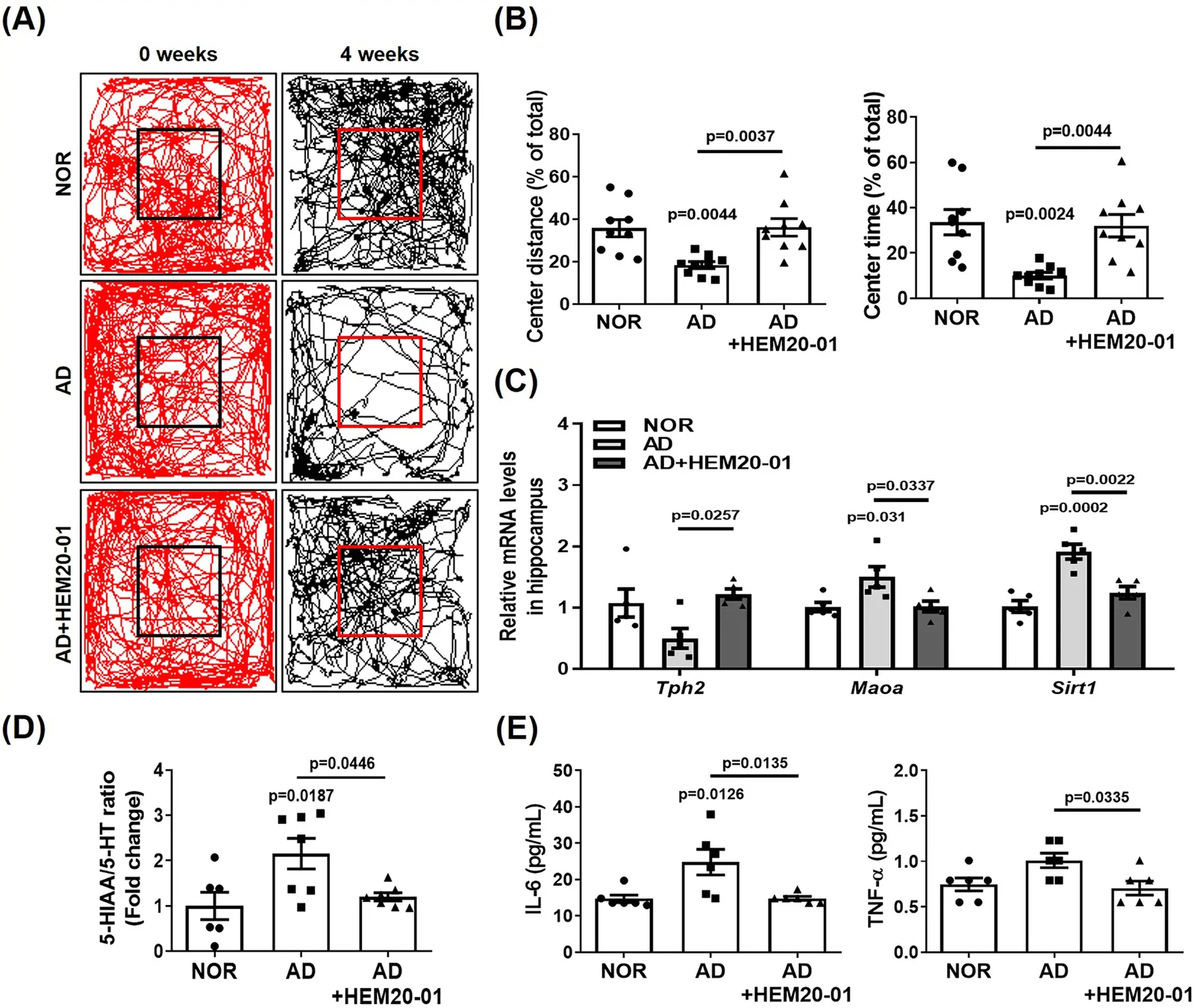
Behavioral and hippocampal serotonergic alterations in HEM20-01-treated mice. **(A)** Representative movement traces from the open field test performed at baseline (week 0) and 4 weeks in each group. **(B)** Distance travelled in the center zone, expressed as a percentage of the total distance travelled, and time spent in the center zone, expressed as a percentage of the total test duration, at week 4 (*n* = 9 per group). **(C)** Relative mRNA expression of *Tph2*, *Maoa*, and *Sirt1* (*n* = 5 per group) and **(D)** 5-HIAA/5-HT ratio (*n* = 6–7 per group) in the hippocampus. **(E)** Multiplex cytokine analysis of inflammatory cytokines in the plasma (*n* = 6 per group). **(B–E)** Data are presented as mean ± SEM and analyzed using one-way ANOVA with Tukey’s test. *P*-values are shown.

AD-like mice also exhibited altered hippocampal serotonergic signatures, including decreased *Tph2* expression, increased *Maoa* and *Sirt1* expression, and an increased 5-hydroxyindoleacetic acid (5-HIAA)/5-hydroxytryptamine (5-HT) ratio, indicating increased serotonin turnover; these alterations were attenuated in HEM20-01-treated mice (**Figures 3C, D**). In addition, circulating TNF-α and IL-6, cytokines reported as depression-associated inflammatory markers in a meta-analysis (19), were elevated in AD-like mice but lower in HEM20-01-treated mice (**Figure 3E**).

### 3.4 Putative succinate-associated taxa and circulating succinate are lower in HEM20-01-treated mice

To investigate whether HDM-induced dermatitis and HEM20–01 treatment are accompanied by differences in gut microbial composition, we performed fecal 16S rRNA gene amplicon sequencing, followed by β-diversity and taxonomic analyses. Principal coordinates analysis (PCoA) based on both Jaccard (presence/absence-based) and Bray–Curtis (abundance-weighted) dissimilarities revealed distinct clustering by group, indicating differences in β-diversity among NOR, AD, and AD+HEM20–01 mice (Jaccard: PCo1 12.2%, PCo2 9.3%; Bray–Curtis: PCo1 17.1%, PCo2 12.9%). Permutational multivariate analysis of variance (PERMANOVA) confirmed significant differences in overall community composition across groups (*P* = 0.001 for both metrics) (**Figure 4A**).

**Figure 4:**
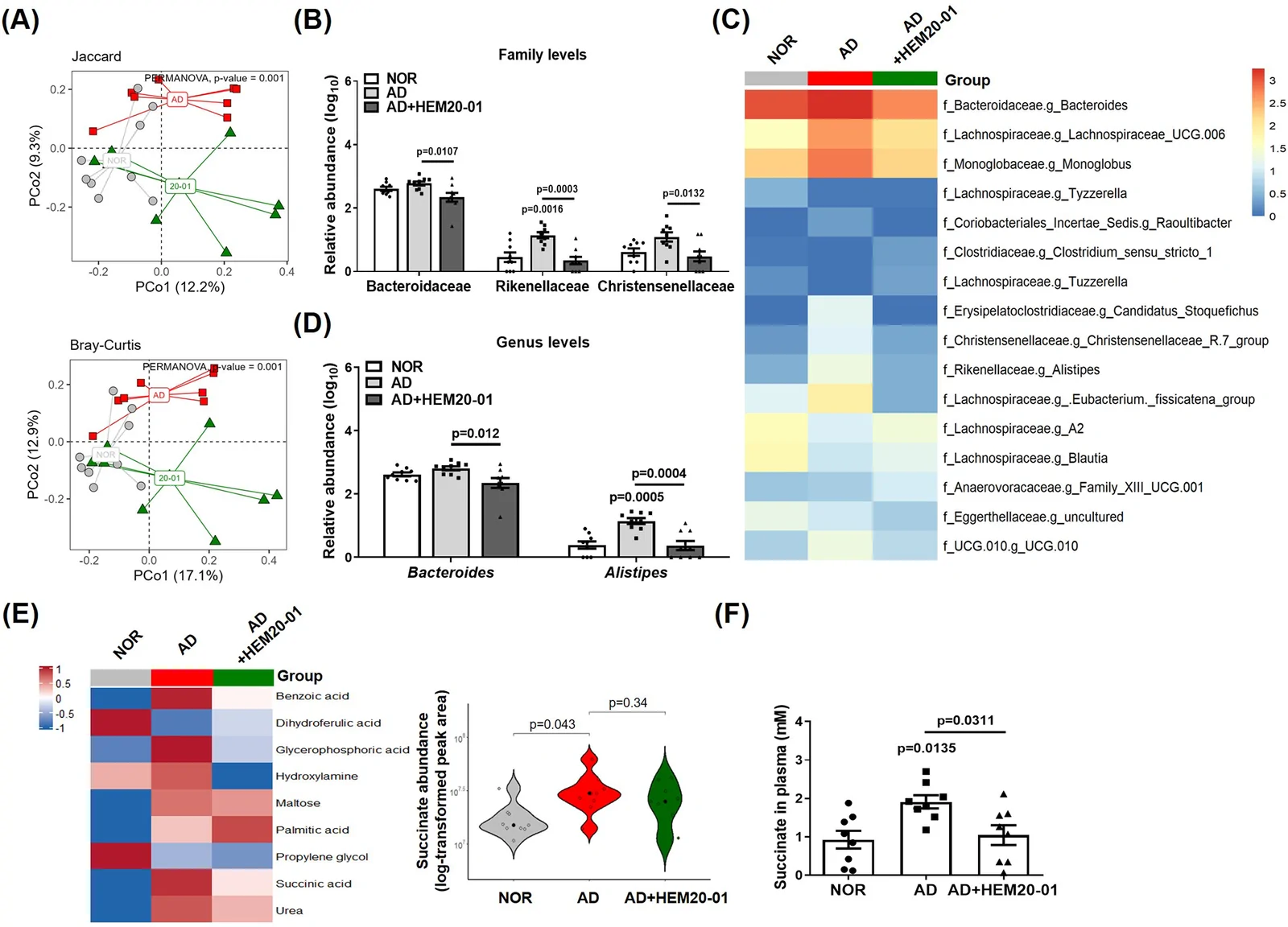
HEM20–01 treatment is accompanied by differences in putative succinate-associated gut taxa and succinate levels. **(A)** PCoA plots based on Jaccard and Bray–Curtis distance metrics of fecal microbial communities (*n* = 9 per group). PERMANOVA *P*-values for overall group differences are indicated for each distance metric. **(B)** Relative abundance of putative succinate-associated bacterial families (*n* = 9 per group). **(C)** Heatmap showing genus-level gut microbial composition based on 16S rRNA sequencing analysis. **(D)** Relative abundance of putative succinate-associated bacterial genera (*n* = 9 per group). **(E)** Fecal metabolomic profiling and succinate quantification (*n* = 9 per group). **(F)** Plasma succinate levels (*n* = 8 per group). **(B, D, F)** Data are presented as mean ± SEM and analyzed using one-way ANOVA with Tukey’s test. **(E)** Data are presented as violin plots showing the distribution of individual values, with median and IQR. Statistical significance was assessed using the Kruskal–Wallis rank-sum test followed by pairwise exact Wilcoxon rank-sum tests with Benjamini–Hochberg (FDR) correction. *P*-values are shown.

Taxonomic profiling of putative succinate-associated taxa showed that Rikenellaceae and its genus *Alistipes* were enriched in AD-like mice compared with NOR mice (*P* = 0.0016 and *P* = 0.0005, respectively), whereas Bacteroidaceae, Christensenellaceae, and *Bacteroides* did not differ significantly between these groups. In contrast, all five of these taxa showed a lower relative abundance in HEM20-01-treated mice than in AD-like mice (**Figures 4B–D**).

To determine whether these microbial composition differences were accompanied by altered metabolite profiles, we next performed fecal metabolomic profiling. Fecal succinate was higher in AD-like mice than in NOR mice (*P* = 0.043) and was lower in HEM20-01-treated mice, although this difference was not statistically significant (*P* = 0.34) (**Figure 4E**). Notably, circulating succinate concentrations were elevated in AD-like mice and lower in HEM20-01-treated mice (*P* = 0.0135 and *P* = 0.0311, respectively) (**Figure 4F**). Moreover, circulating succinate levels were positively correlated with the expression of inflammatory genes in both the skin and intestine (**Supplementary Figures 2A, B**).

### 3.5 Succinate supplementation aggravates cutaneous and intestinal inflammation in MC903-induced dermatitis

Whether succinate exacerbates dermatitis-like inflammatory responses was evaluated in an independent MC903-induced dermatitis model. Mice treated topically with MC903 received 100 mM succinate in the drinking water beginning 2 weeks before MC903 induction and continuing throughout the 3-week induction period (EtOH, MC903+H_2_O, and MC903+succinate groups). Compared with the MC903+H_2_O group, succinate-supplemented mice showed more pronounced epidermal scaling and increased ear thickness, although scratching behavior did not differ significantly between these groups (**Figures 5A–C**). Body weight did not differ significantly among the experimental groups throughout the study (**Supplementary Figure 3**). In the skin, succinate supplementation increased the expression of type 2 inflammation-related genes, including *Il13*, the eosinophil-recruiting chemokines *Ccr3* and *Ccl11*, and the epithelial alarmins *Tslp* and *Il25*, compared with the MC903+H_2_O group (**Figure 5D**).

**Figure 5:**
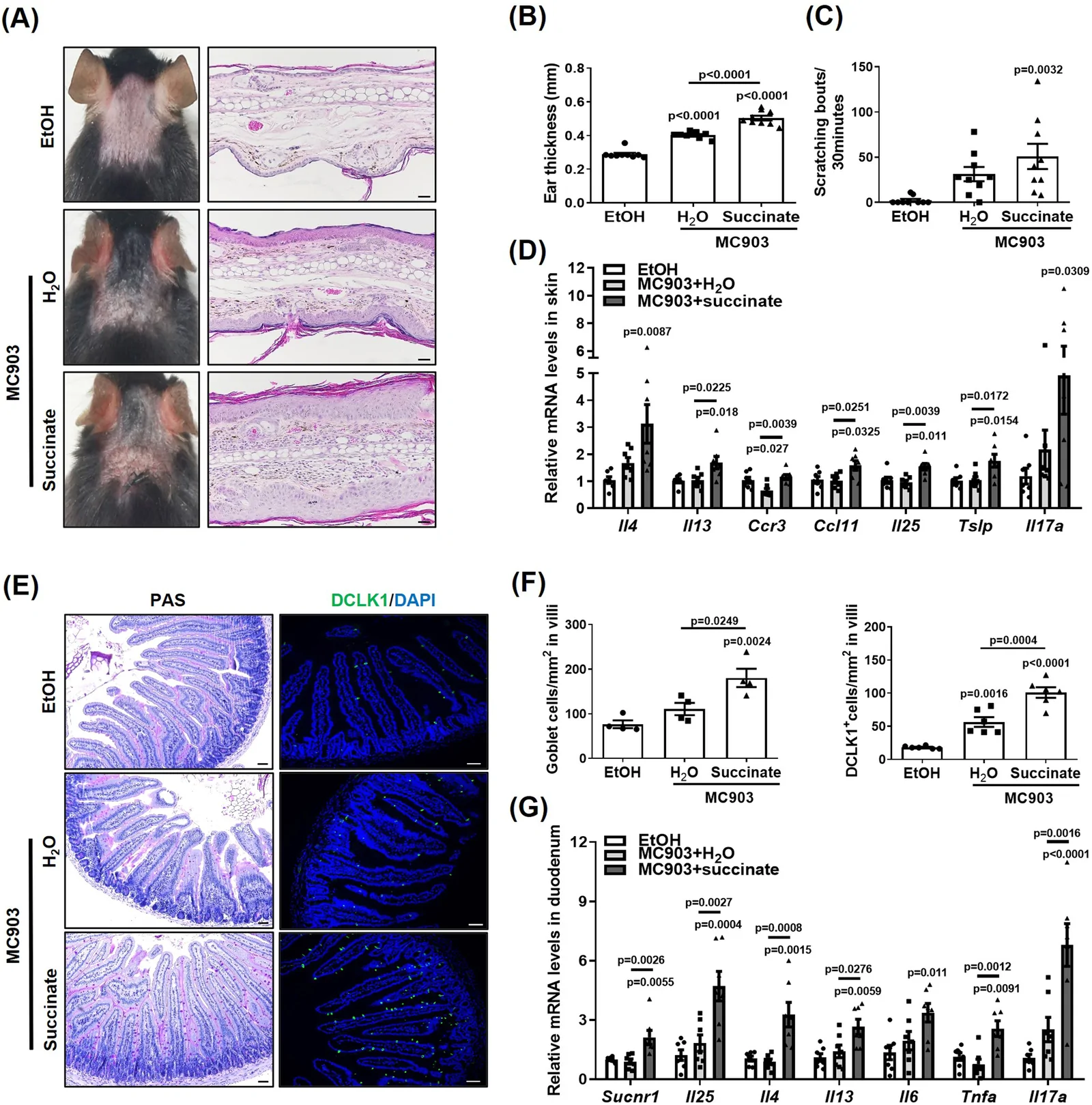
Succinate supplementation aggravates cutaneous and intestinal inflammation. C57BL/6 mice received 100 mM sodium succinate in the drinking water for 2 weeks prior to MC903-induced dermatitis on both ears. **(A)** Representative images of skin lesions and H&E staining in the ear skin. Scale bars, 50 μm. **(B)** Ear thickness and **(C)** scratching behavior (*n* = 9 per group). **(D)** mRNA expression of inflammatory genes in the skin (*n* = 7 per group). **(E)** Representative images of PAS and DCLK1 expression in the duodenum. Nuclei were counterstained with DAPI. Scale bars, 50 μm. **(F)** Quantification of goblet cells (*n* = 4 per group) and DCLK1^+^ cells (*n* = 6 per group) located within villi. **(G)** mRNA levels of epithelial and inflammatory markers (*n* = 7 per group). **(B–D, F, G)** Data are presented as mean ± SEM and analyzed using one-way ANOVA with Tukey’s test. *P*-values are shown.

In the duodenum, succinate supplementation markedly increased goblet and tuft cell numbers without overt disruption of villus-crypt architecture and was accompanied by upregulation of *Sucnr1* and *Il25* (**Figures 5E–G**; **Supplementary Figure 4**). These duodenal changes were accompanied by increased expression of type 2 cytokines (*Il4, Il13*), *Tnfa*, and *Il17a* compared with the MC903+H_2_O group (**Figure 5G**).

Succinate-supplemented mice showed lower center-zone exploration in the OFT than EtOH-treated controls, as indicated by decreases in center-zone distance, center-zone time, and the number of center-zone entries, although these measures did not differ significantly from the MC903+H_2_O group (**Supplementary Figures 5A–D**). Hippocampal expression of *Maoa* and *Sirt1* was also increased (**Supplementary Figure 5F**). However, total distance travelled was lower in succinate-supplemented mice than in EtOH-treated controls (*P* = 0.0023; **Supplementary Figure 5E**), and the group differences in center-zone distance, time, and entries were no longer significant after adjustment for total distance travelled (ANCOVA, *P* = 0.24, 0.47, and 0.65, respectively). These open field test differences were therefore interpreted as activity-related changes rather than as an independent behavioral phenotype.

## 4 Discussion

In this study, we showed that preventive oral administration of HEM20–01 attenuated the development of HDM-induced cutaneous inflammatory phenotypes and was associated with reduced intestinal and behavioral alterations. These differences were accompanied by a lower abundance of putative succinate-associated taxa, lower circulating succinate levels, reduced activation of the intestinal SUCNR1–tuft cell–IL-25 axis, and attenuated hippocampal serotonergic changes (**Figure 6**). In addition, in an independent MC903-induced dermatitis model, succinate supplementation aggravated cutaneous and intestinal inflammation.

**Figure 6:**
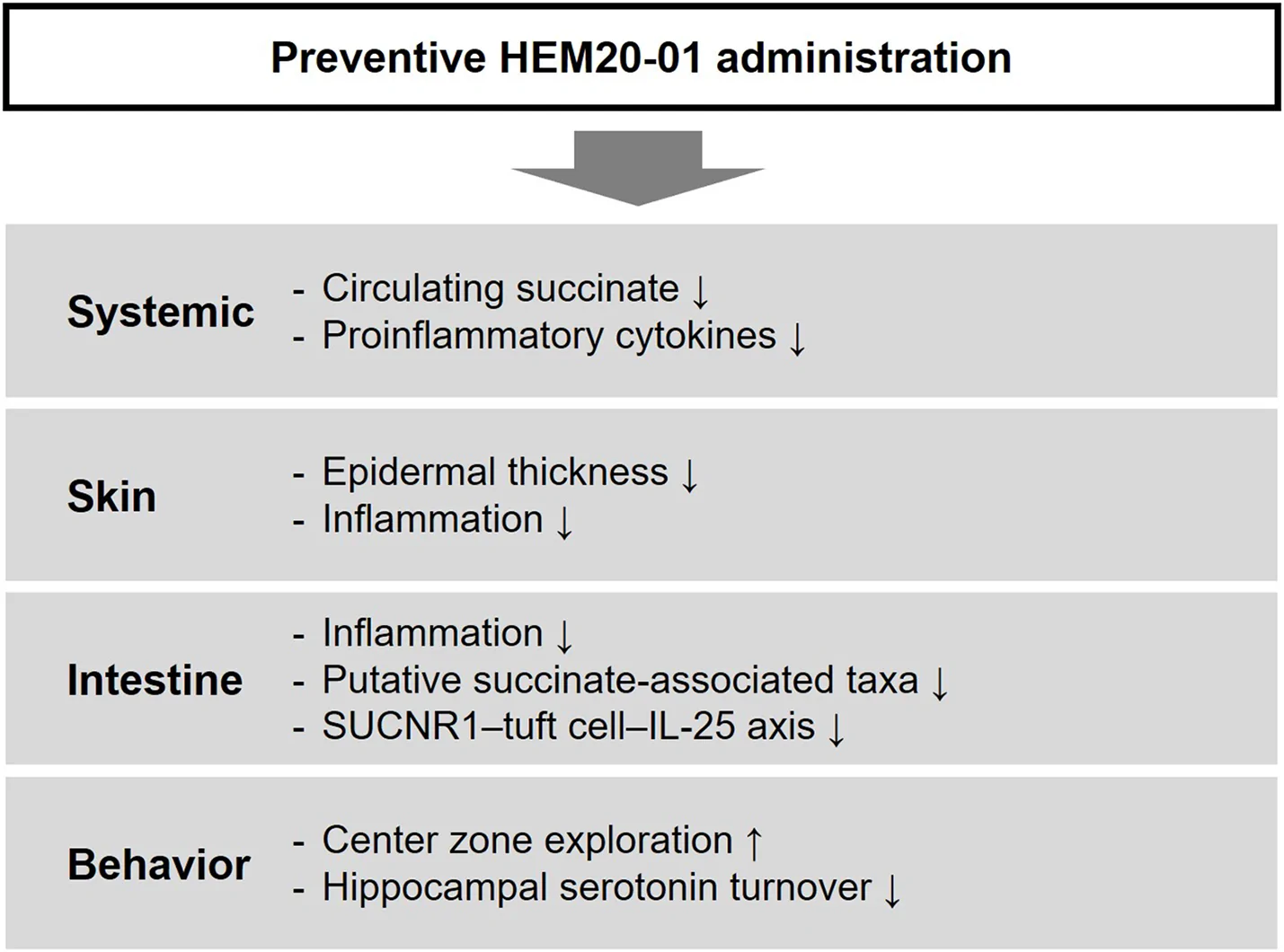
Summary of differences observed in HEM20-01-treated mice. Preventive HEM20–01 administration was associated with systemic, cutaneous, intestinal, and behavioral differences relative to untreated AD-like mice. Downward and upward arrows denote lower and higher values, respectively, compared with untreated AD-like mice.

Succinate has recently emerged as a ‘metabolic alarmin’ that signals through SUCNR1 to amplify immune responses (11, 20). Whereas succinate is normally kept low by cross-feeding bacteria, dysbiotic expansion of succinate-producing taxa can promote its accumulation and systemic exposure, thereby exacerbating inflammation (12–14, 21). In our study, fecal microbiome analysis of HDM-induced AD-like mice revealed expansion of Rikenellaceae and its genus *Alistipes*. These taxa, together with Bacteroidaceae, *Bacteroides*, and Christensenellaceae, have been associated with intestinal succinate metabolism (22–25), and the relative abundance of all five was lower in HEM20-01-treated mice than in AD-like mice. In addition, HEM20-01-treated mice showed lower circulating succinate levels. This finding is consistent with previous reports showing that *Bifidobacterium* species can suppress the expansion of opportunistic or dysbiotic taxa, such as *Bacteroides*, through nutrient competition or modulation of the luminal environment (26–28). In particular, *Bifidobacterium*-derived short-chain fatty acids, such as acetate and lactate, have been reported to lower the luminal pH, thereby creating an environment that is less favorable for acid-sensitive taxa such as *Bacteroides* (27, 29). In humans, the abundance of *Bifidobacterium* has been reported to be inversely correlated with intestinal succinate levels in healthy full-term infants (30). However, whether these microbial and metabolic differences represent direct effects of HEM20–01 or are secondary to the attenuated development of AD-like inflammation requires further investigation.

Elevated succinate can drive intestinal type 2 inflammation through activation of the SUCNR1–tuft cell–IL-25 axis (14–16). In addition, succinate engages broader pro-inflammatory signaling: intracellular succinate stabilizes HIF-1α to promote IL-1β production, while extracellular succinate–SUCNR1 signaling can elicit the release of cytokines such as IL-6 (31–33), consistent with the elevated circulating IL-6 observed in our model. In line with the expansion of putative succinate-associated taxa, HDM-induced AD-like mice exhibited increased circulating succinate levels, together with tuft cell expansion, IL-25 upregulation, and enhanced inflammatory responses in both the skin and gut. These succinate-associated changes were attenuated in HEM20-01-treated mice, and circulating succinate levels correlated positively with inflammatory gene expression in both tissues. Notably, the difference between AD and HEM20-01-treated mice was significant for circulating but not fecal succinate. Fecal levels reflect the local balance between microbial production and cross-feeding consumption, whereas circulating levels integrate intestinal absorption and systemic handling. Therefore, a modest shift in luminal production may alter systemic exposure without a detectable change in fecal content, although limited sensitivity or statistical power of the fecal measurement cannot be excluded. In an independent MC903-induced dermatitis model, oral succinate supplementation further aggravated cutaneous and intestinal inflammatory responses, indicating that exogenous succinate can exacerbate dermatitis-like inflammation.

The high prevalence of anxiety and depression in AD patients has been consistently reported (34–36). Although the mechanisms underlying these emotional symptoms remain incompletely understood, chronic systemic inflammation has been proposed as a potential contributor (37). Chronic systemic inflammation is known to disrupt neural circuits (38, 39), and proinflammatory cytokines can affect the synthesis, release, and reuptake of neurotransmitters such as serotonin, thereby perturbing serotonergic homeostasis (40, 41). Emerging evidence also suggests that succinate may modulate neuroinflammatory responses through SUCNR1 signaling in microglia (42). In addition, elevated circulating proinflammatory cytokines and reduced succinate levels have been reported in patients with major depressive disorder (19, 43). In this study, HDM-induced AD-like mice exhibited reduced center-zone exploration accompanied by dysregulated hippocampal serotonin turnover and increased circulating TNF-α and IL-6 levels, all of which were attenuated in HEM20-01-treated mice. These findings raise the possibility that the behavioral differences observed in HEM20-01-treated mice may be related, at least in part, to the lower systemic inflammatory burden. Further studies are required to determine whether these behavioral differences are secondary to reduced overall disease severity or involve metabolic signals such as circulating succinate.

Several limitations of this study should be considered. First, although our data implicate the intestinal SUCNR1–tuft cell–IL-25 axis, we did not demonstrate that the protective effects of HEM20–01 are causally dependent on succinate reduction; SUCNR1 antagonists or SUCNR1-deficient mice will be required in future studies. Second, we could not determine whether HEM20–01 directly metabolizes succinate or indirectly alters community-level succinate production and utilization, nor whether it stably colonized the intestine; strain-specific metabolic assays and strain-level detection after a washout period will be needed. Third, as mice were co-housed, cage effects on microbial composition cannot be excluded. Fourth, behavior was assessed by the open field test alone, which cannot distinguish anxiety-like behavior from disease-associated changes in activity or discomfort; additional behavioral paradigms will be required to characterize these effects more comprehensively. Finally, validation in human samples will be necessary to establish the translational relevance of our findings.

In conclusion, our study shows that preventive HEM20–01 administration attenuates the development of HDM-induced AD-like inflammatory phenotypes and is accompanied by differences in gut microbial composition, lower circulating succinate levels, and reduced intestinal and behavioral alterations. In addition, exogenous succinate aggravated cutaneous and intestinal inflammatory responses in a separate MC903-induced dermatitis model. Collectively, these findings implicate circulating succinate in AD-associated inflammation and show that preventive HEM20–01 administration attenuates this phenotype.

## Data Availability

Data supporting the findings of this study are included within the article and its **Supplementary Materials**. Raw DNA sequencing data have been deposited in the NCBI Sequence Read Archive under BioProject accession number PRJNA1419489.
