# Supplementary material for: Preventive administration of Bifidobacterium animalis subsp. lactis HEM20–01 attenuates the development of atopic dermatitis-like inflammation in mice, along with gut microbial and succinate alterations
Source: Front Immunol. 2026 Oct 13;17:1915780. doi: 10.3389/fimmu.2026.1915780 (PMC13709991; doi:10.3389/fimmu.2026.1915780)
Supplement: Supplementary file 1 [file Supplementaryfile1.docx]

***Supplementary Material***

**1. Supplementary Figures and Tables**

**1.1. Supplementary Figures**

**Supplementary Figure 1. Center zone entries and total distance travelled.** (A) The number of entries into the center zone and (B) total distance travelled during the open field test at week 4 (n = 9 per group). Data are presented as mean ± SEM. Statistical significance was determined by one-way ANOVA followed by Tukey's multiple comparisons test. *P*-values are shown.

**Supplementary Figure 2. Correlation between plasma succinate levels and inflammatory gene expression.** (A) Correlation between plasma succinate concentrations and the expression of inflammatory cytokine genes in the skin. (B) Correlation between plasma succinate concentrations and the expression of epithelial and inflammatory marker genes in the duodenum. Pearson’s correlation coefficients (R) and *P*-values are shown.

**Supplementary Figure 3. Body weight during the succinate supplementation experiment.** Body weight was measured at −2, 0, and 3 weeks in each group (*n* = 9 per group). Data are presented as mean ± SEM and analyzed using two-way repeated-measures ANOVA followed by Tukey’s multiple comparisons test.

**Supplementary Figure 4. Representative H&E images of the duodenum.** Representative H&E images of the duodenum from each group. Scale bars, 50 μm.

**Supplementary Figure 5. Open field test and hippocampal gene expression after succinate supplementation.** (A) Representative movement traces from the open field test performed at 0 weeks and 3 weeks in each group. Quantification of (B) the percentage of distance traveled in the center zone, (C) percentage of time spent in the center zone, (D) the number of center-zone entries, and (E) total distance travelled is shown (*n* = 9 per group). (F) mRNA levels of *Maoa* and *Sirt1* in the hippocampus (*n* = 7 per group). Data are presented as mean ± SEM and analyzed using one-way ANOVA with Tukey’s test. *P*-values shown in (B–E) are for the comparison with the EtOH-treated control group.

**1.2. Supplementary Tables**

**Supplementary Table 1. Primer sequences for qRT-PCR**

| Gene | Forward (5’–3’) | Reverse (5’–3’) |
| --- | --- | --- |
| ***Gapdh*** | AACTTTGGCATTGTGGAAGG | ACACATTGGGGGTAGGAACA |
| ***Il4*** | ACAGGAGAAGGGACGCCATG | GAAGCCCTACAGACGAGCTCA |
| ***Il13*** | CCTGGCTCTTGCTTGCCTT | GGTCTTGTGTGATGTTGCTCA |
| ***Ccr3*** | CCACTGTACTCCCTGGTGTTCA | GGACAGTGAAGAGAAAGAGCAGG |
| ***Ccl11*** | TCCATCCCAACTTCCTGCTGCT | CTCTTTGCCCAACCTGGTCTTG |
| ***Tslp*** | GCAAATCGAGGACTGTGAGAGC | TGAGGGCTTCTCTTGTTCTCCG |
| ***Il25*** | TGGCTGAAGTGGAGCTCTGCAT | CCCGATTCAAGTCCCTGTCCAA |
| ***Il33*** | CTACTGCATGAGACTCCGTTCTG | AGAATCCCGTGGATAGGCAGAG |
| ***Il1b*** | GGCTGGACTGTTTCTAATGC | ATGGTTTCTTGTGACCCTGA |
| ***Il17a*** | CTCCAGAAGGCCCTCAGACTAC | GGGTCTTCATTGCGGTGG |
| ***Il6*** | AGTCCGGAGAGGAGACTTCA | ATTTCCACGATTTCCCAGAG |
| ***Tnfa*** | GAGTCCGGGCAGGTCTACTTT | CAGGTCACTGTCCCAGCATCT |
| ***Sucnr1*** | CCATCTCTGACTTTGCTTTCCTG | GTGTAGAGGTTGGTGTGAAGCAC |
| ***Prg2*** | CAAGACCTGTCGCTACCTCCTA | GCGGACTGGATTCCGAAGTTAAC |
| ***Tph2*** | CCACCATTGTGACCCTGAATCC | ATGAGGACTCGGTGAGAGCATC |
| ***Maoa*** | GGCTGTCATCAAGTGCATGGTG | GCAGGCATTGACCCATCTGGTT |
| ***Sirt1*** | GGAGCAGATTAGTAAGCGGCTTG | GTTACTGCCACAGGAACTAGAGG |

**2. Supplementary Methods**

**Supplementary Methods 1. Fecal DNA extraction and 16S rRNA gene amplicon sequencing**

Bacterial genomic DNA was extracted from fecal samples using the Mag-Bind® Universal Pathogen Kit (Omega Bio-tek, Norcross, GA, USA) according to the manufacturer’s instructions. Briefly, samples were suspended in 275 μL SLX-Mlus buffer and mechanically disrupted by bead beating using a Mixer Mill MM400 (Retsch, Haan, Germany). Subsequent isolation, washing, and elution steps were performed following the kit protocol.

The V3–V4 hypervariable region of the 16S rRNA gene was amplified using primers 341F (5′-TCGTCGGCAGCGTCAGATGTGTATAAGAGACAGCCTACGGGNGGCWGCAG-3′) and 805R (5′-GTCTCGTGGGCTCGGAGATGTGTATAAGAGACAGGACTACHVGGGTATCTAATCC-3′). PCR was performed using 2× KAPA HiFi HotStart ReadyMix (Roche, Basel, Switzerland) under the following cycling conditions: 95°C for 3 min; 25 cycles of 95°C for 30 s, 55°C for 30 s, and 72°C for 30 s; and a final extension at 72°C for 5 min. Amplicons were purified using HiAccuBead (AccuGene, Incheon, Republic of Korea) and a magnetic stand. Index PCR was performed using IDT indexing primers (Integrated DNA Technologies, Coralville, IA, USA) for the Illumina MiSeq System with 2× KAPA HiFi HotStart ReadyMix under the following conditions: 95°C for 3 min; 8 cycles of 95°C for 30 s, 55°C for 30 s, and 72°C for 30 s; and a final extension at 72°C for 5 min. Libraries were purified, quantified using Qubit 4.0 with the 1× dsDNA HS assay (Thermo Fisher Scientific, Waltham, MA, USA), and sequenced on the Illumina MiSeq platform (Illumina, San Diego, CA, USA). All sequencing experiments were conducted at HEM Pharma Inc. (Suwon, Republic of Korea).

**Supplementary Methods 2. Microbiome bioinformatics and statistical analysis**

Amplicon sequencing data were processed using QIIME2 (v2021.2) (1). Raw fastq.qz files were demultiplexed and quality-filtered using the q2-demux plugin. Denoising, amplicon sequence variant (ASV) inference, and chimera removal were performed using DADA2 via the q2-dada2 plugin (2). Multiple sequence alignment of ASVs was generated using MAFFT (3), followed by construction of a phylogenetic tree using FastTree2 (4) via the q2-phylogeny plugin. Taxonomic assignment was performed using a Naïve Bayes classifier trained on the SILVA 138 99% reference database and implemented with the classify-sklearn method in the q2-feature-classifier plugin (5).

For diversity and abundance analyses, the ASV table was rarefied (subsampled without replacement) to 8,000 sequences per sample. Beta diversity was assessed using principal coordinate analysis (PCoA) based on Jaccard and Bray–Curtis distances. Downstream beta diversity analyses and visualization were performed in R using the phyloseq package (v1.46.0) (6). Group differences in community composition were tested by PERMANOVA (adonis, vegan) with 999 permutations using Jaccard and Bray–Curtis distance matrices. Relative abundance was represented as log-transformed rarefied counts after adding a pseudocount of 1 to each feature count. Selected taxa were chosen a priori on the basis of their reported association with succinate metabolism. Differences in selected bacterial taxa were analyzed using one-way ANOVA followed by Tukey’s multiple-comparisons test.

**Supplementary Methods 3. Sample extraction and untargeted GC–TOF–MS–based metabolomics**

Approximately 50 mg of fecal sample was transferred to a 2 mL microcentrifuge tube and extracted with 50% methanol at a ratio of 1:20 (w/v). Ceramic beads were added to enhance extraction efficiency. Samples were vortexed for 10 min, sonicated for 10 min at room temperature, and centrifuged at 13,000 rpm for 10 min at 4°C. Supernatants were filtered through a 0.2-μm PTFE syringe filter and dried completely using a speed vacuum concentrator (Labogene, Daejeon, Republic of Korea).

For derivatization, dried residues were incubated with 40 μL methoxyamine hydrochloride in pyridine (20 mg/mL) at 30°C for 120 min, followed by incubation with 40 μL N-methyl-N-(trimethylsilyl) trifluoroacetamide (MSTFA) at 37°C for 60 min. Metabolic profiling of feces samples was performed using an Agilent 7890 gas chromatography system (Agilent Technologies, Palo Alto, CA, USA) coupled to a PAL-3 autosampler (CTC Analytics AG, Zwingen, Switzerland) and a Pegasus BT TOF-MS system (LECO Corp., St. Joseph, MI, USA). A 1 μL aliquot of derivatized sample was injected into an Rtx-5MS column (30 m × 0.25 mm, 0.25 μm film thickness; Restek Corp., Bellefonte, PA, USA) using helium as the carrier gas at a constant flow of 1.0 mL/min with a split ratio of 10:1. The oven program was: 75°C for 2 min, ramp to 300°C at 15°C/min, and hold at 300°C for 3 min. The inlet and transfer line temperatures were set to 250°C. Electron ionization was performed at −70 eV, and full-scan spectra were acquired over 45–600 m/z.

Raw data were processed using LECO ChromaTOF software (v5.40) and converted to NetCDF format (*.cdf). Peak detection, retention time alignment, and data correction were performed using the Metalign software suite. Relative metabolite abundances were visualized using heat maps, with fold changes normalized to the mean value of each metabolite across samples. Pathway enrichment was performed by mapping identified metabolites to the Kyoto Encyclopedia of Genes and Genomes (KEGG) database. Untargeted metabolomic profiling was performed using GC–TOF–MS. Succinate was identified by matching its EI mass spectral fragmentation pattern to the NIST spectral library and by confirming both its retention time and fragmentation pattern against an in-house spectral library generated using standard compounds. Succinate abundance was assessed based on the aligned peak area and is therefore reported as relative abundance rather than absolute concentration.

**Supplementary Methods 4. Additional notes on data presentation**

For microbiome analyses, the rarefaction depth and diversity metrics were selected to balance sequencing depth across samples while retaining adequate feature representation. For metabolomics, derivatization and chromatographic conditions were optimized for broad coverage of polar and semi-polar metabolites detectable by GC–TOF–MS.

**3. Supplementary References**

1.	Bolyen E, Rideout JR, Dillon MR, Bokulich NA, Abnet CC, Al-Ghalith GA, et al. Reproducible, Interactive, Scalable and Extensible Microbiome Data Science Using Qiime 2. *Nat Biotechnol* (2019) 37(8):852-7. doi: 10.1038/s41587-019-0209-9.

2.	Callahan BJ, McMurdie PJ, Rosen MJ, Han AW, Johnson AJ, Holmes SP. Dada2: High-Resolution Sample Inference from Illumina Amplicon Data. *Nat Methods* (2016) 13(7):581-3. Epub 2016/05/24. doi: 10.1038/nmeth.3869.

3.	Katoh K, Misawa K, Kuma K, Miyata T. Mafft: A Novel Method for Rapid Multiple Sequence Alignment Based on Fast Fourier Transform. *Nucleic Acids Res* (2002) 30(14):3059-66. Epub 2002/07/24. doi: 10.1093/nar/gkf436.

4.	Price MN, Dehal PS, Arkin AP. Fasttree 2--Approximately Maximum-Likelihood Trees for Large Alignments. *PLoS One* (2010) 5(3):e9490. Epub 2010/03/13. doi: 10.1371/journal.pone.0009490.

5.	Bokulich NA, Kaehler BD, Rideout JR, Dillon M, Bolyen E, Knight R, et al. Optimizing Taxonomic Classification of Marker-Gene Amplicon Sequences with Qiime 2's Q2-Feature-Classifier Plugin. *Microbiome* (2018) 6(1):90. Epub 2018/05/19. doi: 10.1186/s40168-018-0470-z.

6.	McMurdie PJ, Holmes S. Phyloseq: An R Package for Reproducible Interactive Analysis and Graphics of Microbiome Census Data. *PLoS One* (2013) 8(4):e61217. Epub 2013/05/01. doi: 10.1371/journal.pone.0061217.
